# The Treatment of Spreading Peritonitis by Drainage, the Fowler Position, and Rectal Instillation of Saline Solution, with Notes of a Second Successful Case

**Published:** 1908-03

**Authors:** C. Hamilton Whiteford


### THE TREATMENT OF SPREADING PERITONITIS BY DRAINAGE, THE FOWLER POSITION, AND RECTAL INSTILLATION OF SALINE SOLUTION, WITH NOTES OF A SECOND SUCCESSFUL CASE.

BY

C. HAMILTON WHITEFORD, M.R.C.S., L.R.C.P.

THE following case resembles in some details a case treated a year ago, and published in the *British Medical Journal* of July 13th, 1907.

In this second case the patient was an unmarried lady, aged 19, who was first seen on October 15th, 1907.

*Previous history.*—General health very good, with the exception of one occasion, two months ago, when there was slight abdominal discomfort, lasting one day, not sufficiently severe to keep the patient in bed.

*Present illness.*—For last fourteen days discomfort in abdomen. For last four days generalised abdominal pain. For last thirty-six hours moderate general tenderness of abdomen. Up to three days ago the bowels had acted daily, but for fourteen days past the relief had not been satisfactory. Three days ago a dose of cascara produced only a slight movement. Yesterday a soap and water enema produced a fair-sized motion. Menstruation regular; last period normal in every way, ceased ten days ago.

*Present condition.*—At 6 p.m. on October 15th the abdomen was slightly distended and moderately tender. No mass palpable. Nil abnormal in lungs. Nil abnormal per rectum. No vomiting, but feels nauseated. Is taking only liquids in form of lemonade, tea and water. Pulse, 100. Temperature, 101.5° F. Leucocytes, 17,000. Seen at 10 a.m. on October 16th, in consultation with Dr. R. H. Clay, the abdomen was found more distended, with tenderness most marked in right iliac region. The patient had passed a poor night because of the abdominal pain, which fomentations had failed to relieve. Diagnosis: Peritonitis, probably originating in the appendix. In view of the increasing severity of the symptoms, taken in conjunction with the leucocyte count, operation was decided on. A hypodermic injection of morphia, gr. $\frac{1}{4}$, was given to relieve pain during the interval between the consultation and operation.

*Operation.*—With the patient under ether, and reclining at an angle of 45°, the abdomen was opened by an oblique incision midway between the umbilicus and right anterior superior spine. Owing to the excessive fatness of the abdominal wall, this incision was prolonged upwards to the costal margin, and downwards and inwards to within one inch of the right pubic spine, the incision measuring eight inches. The peritoneum, with all the viscera below the transverse colon, was acutely inflamed. The small intestines were bright red, the coils loosely glued together by recent lymph. There was no free fluid. The gall-bladder, stomach and duodenum were healthy. Except that they shared in the general injection, the appendix, the uterus and its appendages were normal. The pelvis was drained by one tube in the vagina and another tube in the lower angle of the wound. The pulse rose during the operation from 100 to 150.

*After History.*—At the end of the operation stercoraceous vomiting commenced and lasted four hours. With the patient in the Fowler position and continuous rectal instillation of saline solution for thirty-six hours the pulse improved rapidly, and on the eleventh day was eighty with a normal temperature. The kidneys secreted very freely. Brown stinking fluid oozed from and was sucked out of both tubes. Examination of this fluid showed the presence of the bacillus coli and staphylococcus albus. On the tenth day a stercoral ulcer formed at the site of the upper tube, which, although shortened by one and a half inches, had not been entirely withdrawn because of the persistence of the foul discharge. On the appearance of the liquid fæces the tube was withdrawn, and the opening into the bowel closed in four days. The vaginal tube was removed on the twelfth day. By the twenty-eighth day the suprapubic sinus had firmly healed, the rest of the incision having united per primam. During the first thirty-six hours nine pints of saline solution were absorbed per rectum.

When it had been definitely ascertained that there was no leakage of visceral contents, and that the upper abdomen was uninvolved, treatment resolved itself into drainage of the affected area, preventing infection of the upper abdomen by the use of the Fowler position, and supplying the patient with fluid by continuous rectal instillation of saline solution, supplemented later by drinks of water and milk.

No cause for the peritonitis was found, no search being made among the inflamed and adherent small intestines.

*Comments: Swing for retaining patient in semi-sitting position.*—To a firm pillow are attached, in its long axis, two strips of stout webbing. The strips lie parallel six inches apart. Their ends, which project one foot on either end of the pillow, converge till they meet, and at their junction is inserted a buckle. Two straps, each four feet in length, are attached to the top bar of the bed. If the head of the bed is unusually low these straps must pass from the head of the bed over the top of the bed-rest on which the patient reclines.

It is an advantage to cover the pillow with mackintosh, between the mackintosh and the back of the thighs is placed a folded towel. With the patient reclining on the bed-rest at an angle of 45°, the pillow is placed under the thighs, the surface free from webbing being next the thighs, and is converted into a swing by being attached to the straps which pass from the head of the bed.

The patient is thus in a swing, the seat of which, instead of being under the buttocks, lies against the back of the thighs. This is practically the method of Mayo-Robson and Moynihan ; it is most efficient, and far more comfortable for the patient than a double inclined plane made of wood or other rigid material.

*Apparatus for administering saline solution per rectum.*—This consists of a rectal tube, sufficiently rigid not to kink—Moynihan's flexible pewter tube answers well—attached by four feet of rubber tubing to the reservoir of saline solution, which is placed in a water bath, which stands over, and is kept warm by a spirit lamp.

The saline solution, one and a half drachms to the pint, in which stands a thermometer, is kept at a temperature of 100° to 105° F. The reservoir is raised four to six inches above the level of the lower rectum. The end of the rectal tube lies just above the internal sphincter.

A clip on the rubber tube regulates the flow to a steady drip of drops, at the rate of not more than one pint per hour. Flatus is not infrequently passed via the tube into the reservoir.

*Morphia.*—When, after operation has been decided upon, a delay of a few hours is inevitable, it is cruel not to make the patient moderately comfortable by a hypodermic injection of morphia. Given for this purpose and at this stage, administration of morphia is beneficial, and has the additional advantage of lessening peristalsis, which is one of the chief agents in spreading infection within the peritoneal cavity.

*The Fowler position, etc.*—The object of the Fowler position is to prevent, or if already present, to diminish infection of the upper abdomen, which is far more fatal than an infection below the level of the transverse colon, absorption taking place much more actively through the lymphatics of the diaphragm than through those of the pelvis.

If infection of the upper abdomen has already taken place, the infected fluid is given a chance of gravitating to the pelvis, whose peritoneum is capable of dealing with large amounts of septic material.

It is probably only a small percentage of the infected fluid which finds it way out by the drainage tubes.

Another advantage of this position is that in a patient with a distended abdomen the heart and lungs can work much more easily in the semi-erect than in the recumbent posture. The continuous supply of saline per rectum supplies the patient with the water of which he has been deprived by vomiting and starvation, and causes a copious secretion of peritoneal fluid, which washes the peritoneum from above downwards. Murphy, of Chicago, considers that the copious supply of fluid "reverses the current of the lymphatic stream, so that, instead of absorption taking place from the peritoneal surface, the mouths of the lymphatics pour out fluid which bathes the peritoneum and carries the infection down to the pelvis." The kidneys act freely and perform their share in eliminating the poison.

*The anæsthetic.*—In this, as in most operations, the stimulating action of ether makes it far preferable to chloroform with its depressing effects.

For cases of septic peritonitis ether, given by an expert, is almost ideal.

*The shortness of the operation.*—In these cases heroic treatment such as evisceration and time-consuming procedures are out of place.

If a viscus in the upper abdomen is leaking the perforation must be either closed or shut off. If the infection is limited to the lower abdomen, unless the source of the infection can be quickly shut off or removed, it is inadvisable to do more than drain the infected area either by the loin, above the pubes or per vaginam, or by all these routes.

With free drainage Nature will localise the infection.

A long operation, theoretically perfect, too often results in the death of the patient, whose life might have been saved by the exercise of elementary common sense.

*The importance of keeping the patient in the semi-erect position before, during and after operation.*—A patient with an infection of any region of the abdomen should be at once placed in the semi-sitting posture, and maintained in this position during removal to and from the operation table, during the operation, and during the first days of convalescence. It is illogical to allow a patient with septic peritonitis to be laid flat on a stretcher or to be carried on a stretcher upstairs feet foremost or downstairs head first. A more effectual way of infecting the upper abdomen could not be devised.

Unless this point is attended to there is considerable risk that the efforts of the surgeon to limit the infection to the lower abdomen will be neutralised by the hospital porters.

